# Research on artificial intelligence, machine and deep learning in medicine: global characteristics, readiness, and equity

**DOI:** 10.1186/s12992-025-01128-1

**Published:** 2025-06-08

**Authors:** Doris Klingelhöfer, Markus Braun, Janis Dröge, David A. Groneberg, Dörthe Brüggmann

**Affiliations:** Institute of Occupational, Social and Environmental Medicine, Goethe University Frankfurt, Theodor-Stern-Kai 7, 60590 Frankfurt, Germany

**Keywords:** Medical AI, Research areas, Publication output, Risks, Big data, Global equity

## Abstract

**Background:**

Artificial intelligence (AI) will have a lasting and drastic impact on medical research and healthcare. In addition to the benefits, the associated risks are also the subject of controversial debate and there are fears of serious consequences. There is an urgent need for action, which must be underpinned by scientific information.

**Methods:**

By analyzing temporal and geographic patterns, including national readiness for access to AI, we therefore identified incentives and barriers to global research under socioeconomic conditions.

**Results:**

The explosive increase in annual publications started in 2017. The main players in AI_med_ research were the USA, China, the UK, Germany, and South Korea. There was a significant correlation between the publication output on AI in medicine (AI_med_) and the metrics for economy and innovation. Additionally, citation patterns show the disadvantage of the Global South compared to the North American and European countries. In several weaker economies: Jordan, Pakistan, Egypt, Bangladesh, and Ethiopia, a more positive position was found in relation to the number of articles suggesting a better ranking in AI_med_ research in the future.

**Conclusion:**

The results show the need for advanced global networking to ensure all relevant aspects for equitable development and the beneficial use of AI_med_ without promoting racial or regional inequities and to enforce this not only in the AI systems of economically strong countries but also for the participation of economically weaker countries.

**Supplementary Information:**

The online version contains supplementary material available at 10.1186/s12992-025-01128-1.

## Background

Recently, the *Center for Artificial Intelligence Safety* (CAIS) published an open letter in which many renowned scientists, policymakers, and even *artificial intelligence* (AI) experts, such as the CEOs of *OpenAI* Sam Altman and *Google DeepMind* Demis Hassabis, warned of the risks of AI to humanity. It states, ”Mitigating the risk of extinction from AI should be a global priority alongside other societal-scale risks such as pandemics and nuclear war” [1]. This statement aims to open a discussion that seems urgently needed. In March 2024, the EU passed the Artificial Intelligence Act, which represents the first regulation of AI. Products are categorized according to risk level, and all technologies that pose risks classified as “unacceptable” are banned, e.g. scoring systems or manipulative AI [2]. The rapid development of AI in recent years has revolutionized various fields, including medicine and healthcare.

Numerous application areas of AI have emerged that can accelerate and optimize medical procedures and offer a wide range of potential uses. Those tools are capable of using healthcare big data, such as electronic medical records, medical images, and manual notes, to automate processes or procedures, assist clinicians, and improve and deepen the understanding and causes of complex health disorders [3]. AI can also support pandemic preparedness and response [4]. The application areas of AI range from robotic surgery and transplantation techniques to decision-making and diagnostics, enabling speech and image recognition approaches [5]. In particular, the recognition of complex patterns and identification of anomalies in X-rays, *computer tomography* (CT) scans, and *magnetic resonance imaging* (MRI) enable better and faster diagnosis of cancer, neurological, and coronary diseases [6–8]. Moreover, therapies can be more accurately tailored to patients through individual genetic profiling [9]. AI also plays a role in the development of new drugs. Another area of application is risk management, where it could play a role in decision-making and planning in complex situations [10]. Taking all these forms of application into account, they have the potential to change the healthcare sector [11]. In conjunction with these ever more rapidly developing approaches, certain risks and dangers are emerging that are followed by threats to humanity [12]. There are major concerns about ethics, laws, and safety - and their monitoring - combined with low social acceptance, which hinders the safe and orderly development of AI in medicine. So far, there is a lack of precise, binding instruments that keep pace with the speed of AI development [13, 14], as well as an international standardization of governance for AI in medicine [15]. Standardized processes, the security of patient data, and compliance with privacy regulations are critical. Machines as decision-making tools are questioned in terms of ethical and control mechanisms. They are often not transparent or explainable to medical staff [5]. There are fears of unforeseeable and therefore dangerous consequences in relation to healthcare, such as invasion of privacy, social injustice, racism, dehumanization, lack of informed consent, and accountability for harm [16].

Improving the accuracy of AI is based on extremely large data sets and therefore threatens security, privacy, and confidentiality, especially of individual data [17]. The hacking has increased tremendously. Referring to medical files, governments, and critical infrastructure can be attacked in this way [18]. There is also an ethical distortion of big data. Minorities, ethnic groups, or genders can be significantly misrepresented and, therefore, do not represent the general population [19]. This can also be done intentionally to incorporate bias into healthcare [18]. The introduction of AI in risk management also poses major challenges. Implementing new AI into legacy systems is difficult and the sheer volume of data, which can be inconsistent and of poor quality, can lead to incorrect assessments and therefore wrong, potentially fatal decisions. There are also ethical concerns, as there is a risk of data misuse and security or transparency deficiencies persist. Discriminatory distortions are also possible due to the integration of biased, trained AI models [10].

In addition to the manipulative, controlling, and oppressive dangers of AI, *lethal autonomous weapon systems* (LAWS) can be created without human oversight, e.g., drones equipped with explosives capable of mass destruction [20]. The societal and public health impacts of job displacement, which could lead to mass job losses, must also be considered [21].

Therefore, further development of AI must be accompanied by a deeper understanding of AI, including its implications for societal equity and health security, as well as a deep and broad public and professional debate [14]. Global regulations and guidelines must be developed that are binding to meet the ethical imperatives of avoiding unequal treatment, techniques to curb bias, and the prevention of harmful or misleading information [22]. This requires the identification of weaknesses in the application of AI_med_. This in turn demands the definition of scientifically based political guidelines, the inclusion of weaker economies in global or regional regulations, and the creation of ethnically and regionally balanced values for the generation of all medical AI databases. In particular, the use of large multimodal models (LMMs) with their application across the healthcare system (diagnosis, administration, education, research, patient-led application) may provide false, biased, or incomplete information. Therefore, applications related to race, ethnicity, gender or gender identity, and age may provide poor-quality data and have a discriminatory impact [23].

The development of AI applications takes place in universities and academic health centers with ethical review, but also in companies that do not fall under ethical regulators [24]. The practice of “ethics dumping” to gain approval for researchers from high-income (HI) countries to the low regulations from low-income or middle-income (LMI) countries is evidenced [25] as well as the authorization to use data for commercial purposes [26]. Additionally, LMI countries often do not have access to the data for training AI models.

This highlights the need for strategies for ethically responsible application in the context of global health governance. *Research Ethics Committees* (RECs), also known as *Institutional Review Boards* (IRBs), can play an important role, e.g., in monitoring data sharing, managing AI concepts, or identifying risks for those whose data is included in AI. Researchers’ conflicts of interest must also be taken into account to ensure open-ended research and avoid results being obtained in line with commercial interests [27]. In the USA, however, it has become apparent that the RECs are not sufficiently prepared to meet the current requirements of AI [24]. This makes it clear that the governance instruments need to be adapted and updated to take account of new research contexts [28]. Particularly at the global level, updating the functions of the regional environmental councils is crucial [24]. The distribution of responsibility between all actors is particularly important for global health research that goes beyond national borders. To enforce and monitor ethical regulation at an international level is the responsibility of the regional economic councils and of national governments, international organizations, healthcare professionals, and technology providers.

For this purpose, adequate scientific action is imperative, as there is a crucial need for knowledge and networking. Despite these challenges, AI offers the opportunity to take medicine at a higher level with the participation of AI developers, physicians, clinicians, scientists, policymakers, and ethicists. To provide all these stakeholders with a sound background knowledge of what has been researched in the past, for what reasons, and under what conditions, this study collected, analyzed, and interpreted research activities in the field of medical AI (AI_med_). An in-depth analysis of article metadata will shed light on the AI-Med research landscape and highlight the global patterns of scientific endeavors in this field. This approach will be used to identify urgent needs in the scientific field and determine the responsible parties. Given that a country’s economic indicators and innovation strength likely impact its AI status, analyses in this area will illuminate the relationship between these factors and AI in medicine within publishing countries. Concerning this, a further aim of this study is to analyze the capabilities and readiness of the countries reflected in their publications on AI_med_. The implications for global equity requirements are discussed.

## Methods

### Methodological platform

The methodological platform NewQIS (*New Quality and Quantity Indices in Science*) [29] was used for all analyses in this study. NewQIS was established at the Charité Berlin in 2009 as a bibliometric tool for analyzing research areas in medicine and the life sciences. It combines proven bibliometric approaches with advanced tools. Socio-economic characteristics of the countries of publication are included, allowing for an in-depth assessment of chronological and geographical patterns on specific scientific topics. NewQIS has already served as a methodological platform for a large number of studies [29] as background information for all those involved in the respective field of investigation, ranging from individual scientists to clinicians and political decision-makers. Here, the NewQIS standard methods were adapted to the study objective and further developed to reflect the global research landscape of AI_med_ to identify research incentives, needs, and barriers.

The basic principle of NewQIS is the combination of bibliometric tools and the sophisticated visualization technique of *Density-Equalizing Map Procedures* (DEMP), which is based on a cartogram algorithm developed by Gastner and Newman [30]. Following the physical effect of density compensation, the countries are either enlarged or reduced in size depending on the value of the analyzed parameter. This leads to a distortion of the world map and makes it easier to grasp geographical patterns at a glance.

The *Web of Science Core Collection* (WoS) online literature database was used as a standard data source for all NewQIS studies. The Core Collection provides publications that meet the strict requirements of the WoS and citation counts referring to the *Journal Citation Report* (JCR). It is one of the most established scientific literature databases used for bibliometric approaches.

### Search strategy and data collection

A search strategy must be developed for retrieving the metadata of publications related to the search target to ensure the creation of a representative and comprehensive database. When defining the search terms, we followed the definition of the *Singapore Computer Society* (SCS) [31] of AI. Thus, “deep learning” (DL) is part of “machine learning” (ML), which in turn is a subfield of “artificial intelligence” (AI) with the following definitions: AI: intelligent systems capable of performing tasks that would otherwise require human intelligence; 2) ML: algorithms capable of learning from data and making decisions based on it (with human intervention); and 3) DL: artificial neurological networks are used to make accurate decisions (without human intervention) [31].

Two search strings are combined into a sophisticated strategy (Supp. Table 1):

**Table 1:** Medical research areas. (A) title words assigned to medical disciplines with more than 100 occurrences and the percentage of all title words analyzed (threshold: 5 occurrences). (B) most frequently assigned web of science (WoS) categories of medical disciplines with more than 1000 articles and their percentage share

A) Medical disciplines	Number of title words	%	B) WoS category (Medical disciplines)	Number of articles	%
Radiology / Nuclear medicine	2203	20.75	Radiology, Nuclear Medicine & Medical Imaging	5223	12.64
Oncology / Radiotherapy	1614	15.20	Neurosciences & Neurology	3492	8.45
Health care / Public Health	713	6.72	General & Internal Medicine	2494	6.04
Diagnosis / Prediction	702	6.61	Oncology	2116	5.12
Cardiology	512	4.82	Health Care Sciences & Services	1922	4.65
Ophthalmology	499	4.70	Public, Environmental & Occupational Health	1107	2.68
Epidemiology	490	4.62	Surgery	1103	2.67
Psychiatry / Psychology	489	4.61	Psychology	1103	2.67
Neurology / Neurosciences	478	4.50	Pharmacology & Pharmacy	1081	2.62
Gastroenterology	285	2.68	Cardiovascular System & Cardiology	1011	2.45
Orthopedics / Sports medicine	232	2.19	Genetics & Heredity	748	1.81
Emergency medicine / Intensive Care	205	1.93	Psychiatry	721	1.75
Respiratory system	198	1.87	Ophthalmology	650	1.57
Genetics	181	1.70	Gastroenterology & Hepatology	494	1.20
Gynecology / Obstetrics / Neonatology	161	1.52	Orthopedics	405	0.98
Pharmacology / Drug therapy	151	1.42	Endocrinology & Metabolism	363	0.88
Hepatology	130	1.22	Dentistry, Oral Surgery & Medicine	319	0.77
Biochemistry / Molecular biology	128	1.21	Pathology	277	0.67
Pathology	122	1.15	Immunology	272	0.66
Immunology (cellular / molecular)	114	1.07	Pediatrics	257	0.62
Angiology	112	1.06	Orthopedics	405	0.98

1. Accordingly, the terms “artificial intelligence”, “machine learning”, and “deep learning” were searched for in the title field of WoS. We identified 73 WoS categories on medical research areas (WoS search field Web of Science Categories) (Supp. Table 1) and combined the title search with them.
2. The entries found in this way do not include publications that only contain the abbreviation “AI” in the title. To include these publications, the term “AI” was searched for in the title and combined with the term “Artificial Intelligence” in the “TOPIC” field. The tool “TOPIC” searches the title, the summary, and the keywords of the article. This approach was necessary because the abbreviation “AI” is not only used for artificial intelligence. Therefore, the direct reference must be ensured by searching for this term in the summary or in the keywords of the publications. This approach prevents incorrect entries. This second search sequence was also combined with the WoS categories from the field of medicine (Suppl. Table 1).

### Data processing

For subsequent analyses, the determined metadata of the articles found were stored in an MS Access database. Some of the metadata has to be corrected manually since no standardized entries are provided by the WoS. Thus, the information on the authors’ institutions must be unified and the information on the countries of origin must be updated to be evaluable. The data gathered in this way were sorted according to the parameters to be analyzed, and new values were calculated for further evaluation (Supp. Figure 1).

For the socioeconomic comparison of countries’ performance on AImed, the database of the UNESCO Institute for Statistics (UIS) was used [32]. Only the data for Taiwan were obtained from another source, as the UIS does not provide data [33]. The data used in each case are population size and gross domestic product (GDP). In addition, the *Global Innovation Index* (GII) was used to analyze the countries’ performances. The GII is a ranking that represents the innovation capacity of 132 countries including those for AI applications. It is released annually by the French business school INSEAD, Cornell University, and the UN’s *World Intellectual Property Organization* (WIPO) [34].

Moreover, the *Governmental AI Readiness Index* (GAIRI) was used for the analyses. It was created in 1017 to assess the ability of countries to take advantage of AI and realize its potential. The index combines eleven metrics across governance, infrastructure and data, skills and education, and government and public services [35].

### Analyses

In addition to the established bibliometric analyses, such as chronological trends and country performances, socioeconomic country data were included in the study to better assess the individual contributions of the publishing countries. Thus, ratios between article numbers and population size (in millions of inhabitants) or gross domestic product (GDP in US$10 billion) were calculated. These metrics normalize the publication output of countries according to their demographic and economic characteristics to enable a comparison of publication metrics under the same conditions in terms of population size and GDP. Data from 2021 from the UNESCO Institute for Statistics (UIS) were used for this purpose, as this is the most up-to-date complete dataset that enables a reliable global comparison of publishing countries [32]. In addition, correlation analyses and linear regressions (Spearman) were performed for nonparametric data between the number of articles on AI_med_ and the countries’ GDP and GII scores, respectively. The residuals of both analyses were calculated and visualized.

Network structures were elaborated at the international and institute levels. The assigned WoS categories were used for the analysis of the research foci. The titles and keywords of the articles were identified and analyzed. VOSviewer was used for the identification of titles and keywords as well as subsequent clustering. To enable the manual assignment of title words to various medical disciplines, a threshold of at least five occurrences was established for analysis. Including all title words in the analysis would be too unspecific and contain too many commonly used terms. The definitions of the various medical disciplines for the assignment of title words were based both on the WoS categories and on further medicine-related subject areas such as “diagnostics” and “therapy”. However, a distinct assignment was not always possible due to the interdisciplinary use of terminology. In addition, some of the title words that VOSviewer identified were either too general or were used so variably that they could not be assigned to a specific discipline and were therefore neglected.

To carry out some analyses, it is essential to set threshold values. This applies to all calculations of ratios of country indicators (citation rate, R_GDP_, R_POP_) to avoid a bias in favor of countries with very low publication performance. In addition, some threshold values must be defined for the presentation of the results to obtain meaningful figures (cluster analyses, cooperation analyses).

### Methodological limitations

As with all methods based on scientific literature databases, there are of course limitations to this study. First, not all related publications can be included, as the WoS does not list all scientific journals. Only journals with reputable peer review systems and qualitative standards can be searched. The use of the search term is limited because the strategy always involves balancing the goal of finding as many relevant articles as possible with the need to minimize incorrect entries. Additionally, all citation analyses rely on the citation numbers provided by WoS for each article. Basically, these numbers can be biased by misleading citations, erroneous citations, and citation stacking. Furthermore, the assessment of scientific work quality based on these metrics is limited, as it only reflects the scientific community’s perception, which can occasionally be negative. In addition, all metadata retrieved by WoS may be inaccurate due to incomplete or incorrect entries. This does not affect the validity and representativeness of the generated database.

## Results

The search strategy identified 29,192 original research articles (n) in the WoS Core Collection on AI_med_.

### Research foci

First, based on the text data, an analysis of the words occurring in the titles of the articles was performed. A total of 19,881 title words (tw) that occurred at least five times (threshold) could be identified. Of these, 16,702 words could be assigned to a specific topic in the field of methods and programming (tw = 6086) or medical disciplines and topics (tw = 10,616). By far the most frequently used title words were related to *Medical Imaging* (tw = 2203, 20.75%) and *Oncology / Radiotherapy* (tw = 1617, 15.20%). However, words related to *Health Care / Public health* (tw = 713, 6.72%), *Diagnosis / Prediction* (tw = 702, 6.61%), and *Cardiology* (tw = 512, 4.82%) were also identified at least 500 times. All medical topics that occurred at least 100 times are listed in Table 1A. Second, based on bibliographic data, the WoS categories were evaluated. Again, *Radiology*,* Nuclear Medicine & Medical Imaging* were the most common (*n* = 5223, 12.64%), followed by *Neuroscience & Neurology* (*n* = 3429, 8.45%), *General & Internal Medicine* (2494, 6.04%), *Oncology* (*n* = 2116, 5.12%), and *Health Care Science & Services* (*n* = 1922, 4.65% (Table 1B). Additionally, a cluster analysis of the title words and the keywords was carried out based on the weighted linkages between the two terms. For the title words applied, five main clusters around the main search terms AI (*artificial intelligence*), ML (*machine learning*), DL (*deep learning*) >2, and medical applications can be found. (1) The largest cluster (ML) includes 14,099 occurrences of 68 words related to predictive models of mortality and morbidities. (2) Title words around the DL were identified 13,078 times among the 38 words related to image segmentation. (3) With 8,855 occurrences of 54 word identifications, the AI cluster words are related mainly to research and different study designs of health care projects.

**Fig. 1:**
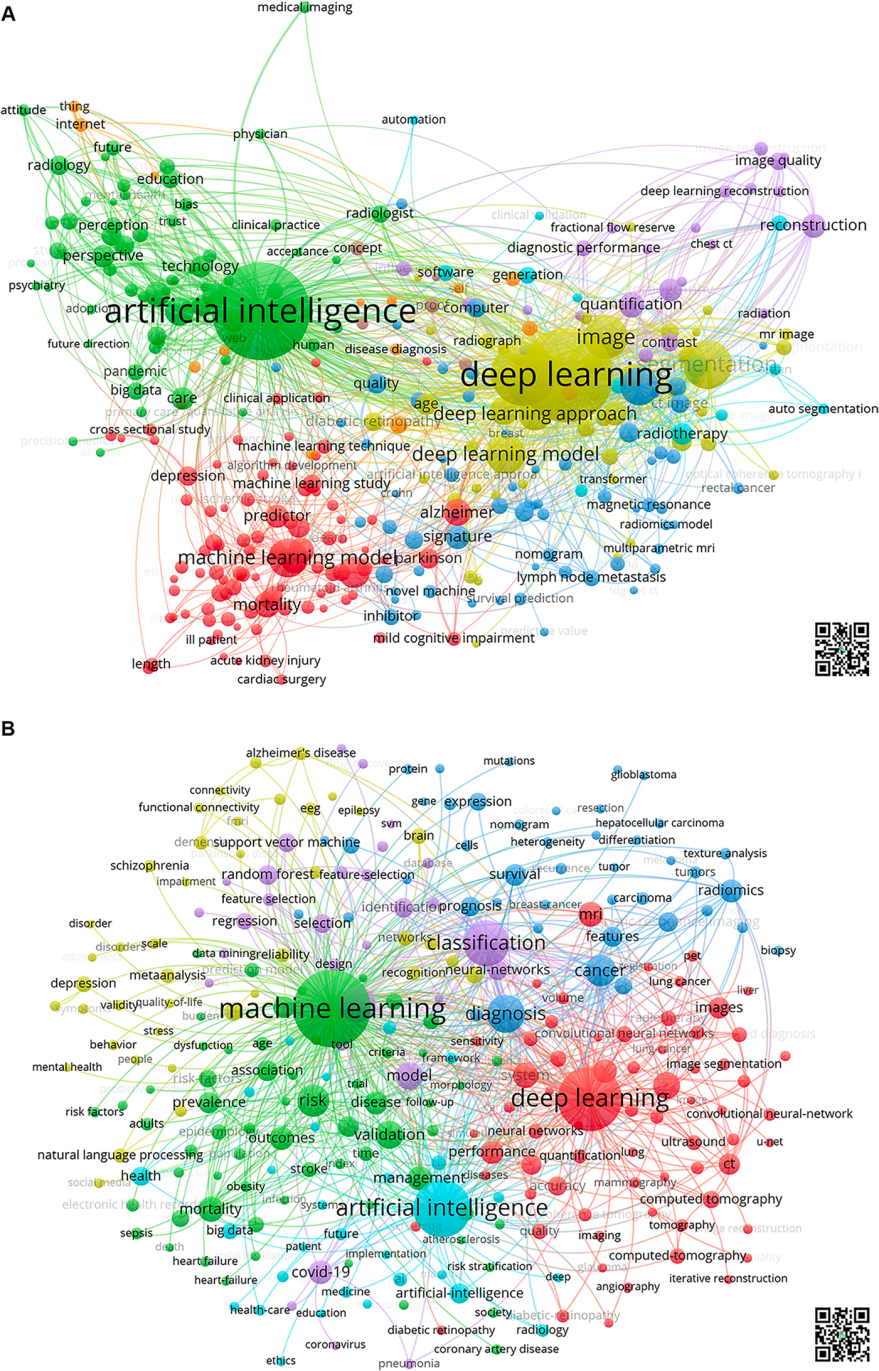
Cluster analysis of **A**) Title words (threshold > 30 occurrences). Red cluster: Machine Learning (For display reasons, “machine”, as the most frequently occurring title word, was not included), yellow cluster: Deep Learning, green cluster: Artificial Intelligence, blue cluster: oncology, violet cluster: radiology, diagnosis. QR code for interactive display. **B**) Keywords (threshold > 100 occurrences), Red cluster: Deep learning, medical imaging, neural networks; green cluster: Machine learning, risk, prediction; light blue cluster: Artificial intelligence, violet cluster: modeling, classification, COVID-19; yellow cluster: psychology, psychiatry, blue cluster: Oncology. QR code for interactive display

With a lower number of occurrences, the other identified clusters are related to 4) oncological and 5) radiological and diagnostic topics (Fig. 1A).

**Fig. 2:**
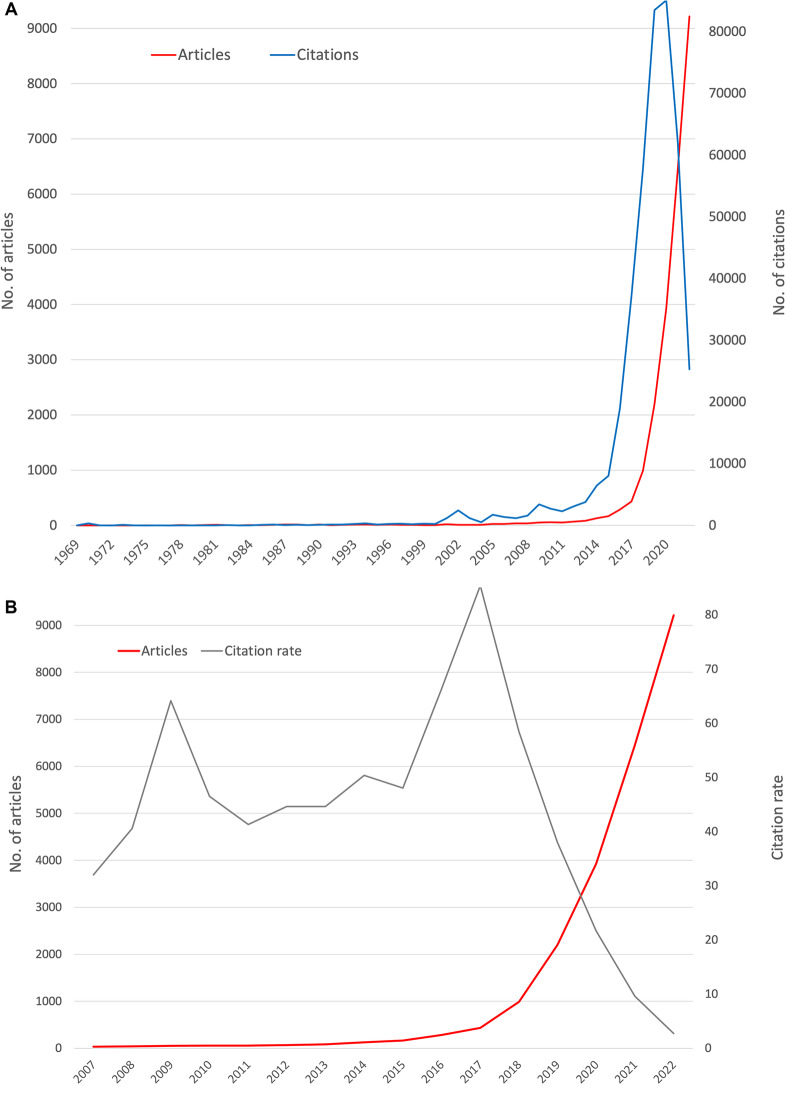
Timely development of AImed articles. **A**) Number of articles and citations from 1969 to 2022, **B**) Number of articles and average citation rate from 2007 to 2022 (years with at least 30 articles on AImed = threshold)

Keyword cluster analysis identified different groups around the three main areas (AI, ML, DL) with various topics (Fig. 1B). These findings also relate to several areas ranging from medical imaging, oncology, psychology, and psychiatry to modeling and classification (COVID-19) and risk assessment and prediction.

### Publication trends

The first article indexed in WoS that met the search criteria was published in 1969. Since then, a few AI_med_ articles have been published almost every year, reaching triple digits only in 2014. Since 2000, however, there has already been a slight increase. The annual publication numbers exploded after a further moderate increase from *n* = 435 in 2017 to *n* = 9218 in 2022. Similarly, the trend in the annual citation numbers (c) for publications on AI_med_ developed, although they reached the beginning of the extreme increase earlier. Since 2015, which has already reached a value of c = 8020, a significant increase can be observed, which peaked at c = 85.517 at the time of the evaluation in 2020. This was followed by a clear decrease to c = 25,269 in 2022 (Fig. 2A). The period of the 2000s showed two peaks in the annually calculated citation rate (cr): 2009 (cr = 64.15) and 2016 / 2017 (cr = 66.34 /85.41) (Fig. 2B).

The most-cited articles of this study are listed in Table 2.

**Table 2:** Most-cited articles on AI_med_, c = number of citations

Authors (Countries)	Year	c	Title	Journal	Funding Agency
G. Litjens et al. [45] (Netherlands)	2017	5616	A survey on deep learning in medical image analysis	Medical Image Analysis	Dutch Cancer Society
V. Gulshan et al. [46] (USA, India)	2016	3212	Development and Validation of a Deep Learning Algorithm for Detection of Diabetic Retinopathy in Retinal Fundus Photographs	JAMA	Google Inc.
M.A. Shipp et al. [68] (USA, UK, Barbados)	2002	1785	Diffuse large B-cell lymphoma outcome prediction by gene-expression profiling and supervised machine learning	Nature Medicine	Bristol-Myers Scibb / Affymetrix
B.E. Bejnordi et al. [47] (Netherlands)	2017	1335	Diagnostic Assessment of Deep Learning Algorithms for Detection of Lymph Node Metastases in Women with Breast Cancer	JAMA	FP7 (EU), Stichting IT Projecten / FES (Netherlands)
R.C. Deo [69] (USA)	2015	1256	Machine Learning in Medicine	Circulation	National Heart, Lung, and Blood Institute (NHLBI/NIH)
A. Mathis et al. [70] (Germany, USA)	2018	1218	DeepLabCut: markerless pose estimation of user-defined body parts with deep learning	Nature Neuroscience	FP7 (EU) / German Science Foundation (DFG), IARPA (MICrONS)
N. Coudray et al. [71] (USA, Greece)	2018	1111	Classification and mutation prediction from non-small cell lung cancer histopathology images using deep learning	Nature Medicine	Laura and Isaac Perlmutter Cancer Center (NYU)
A. Esteva et al. [72] (USA)	2019	1111	A guide to deep learning in healthcare	Nature Medicine	Google Inc.
J. De Fauw et al. [73] (UK)	2018	1068	Clinically applicable deep learning for diagnosis and referral in retinal disease	Nature Medicine	NIHR, Institute of Ophthalmology (UCL) / College of Optometrists (UK)
R. T. Schirrmeister et al. [74] (Germany)	2017	1060	Deep Learning With Convolutional Neural Networks for EEG Decoding and Visualization	Human Brain Mapping	BrainLinks-BrainTools (DFG), Federal Ministry of Education and Research (BMBF)

### Countries of origin

From the total database, *n* = 29,114 articles could be assigned to a country of origin (99.73%).

**Fig. 3:**
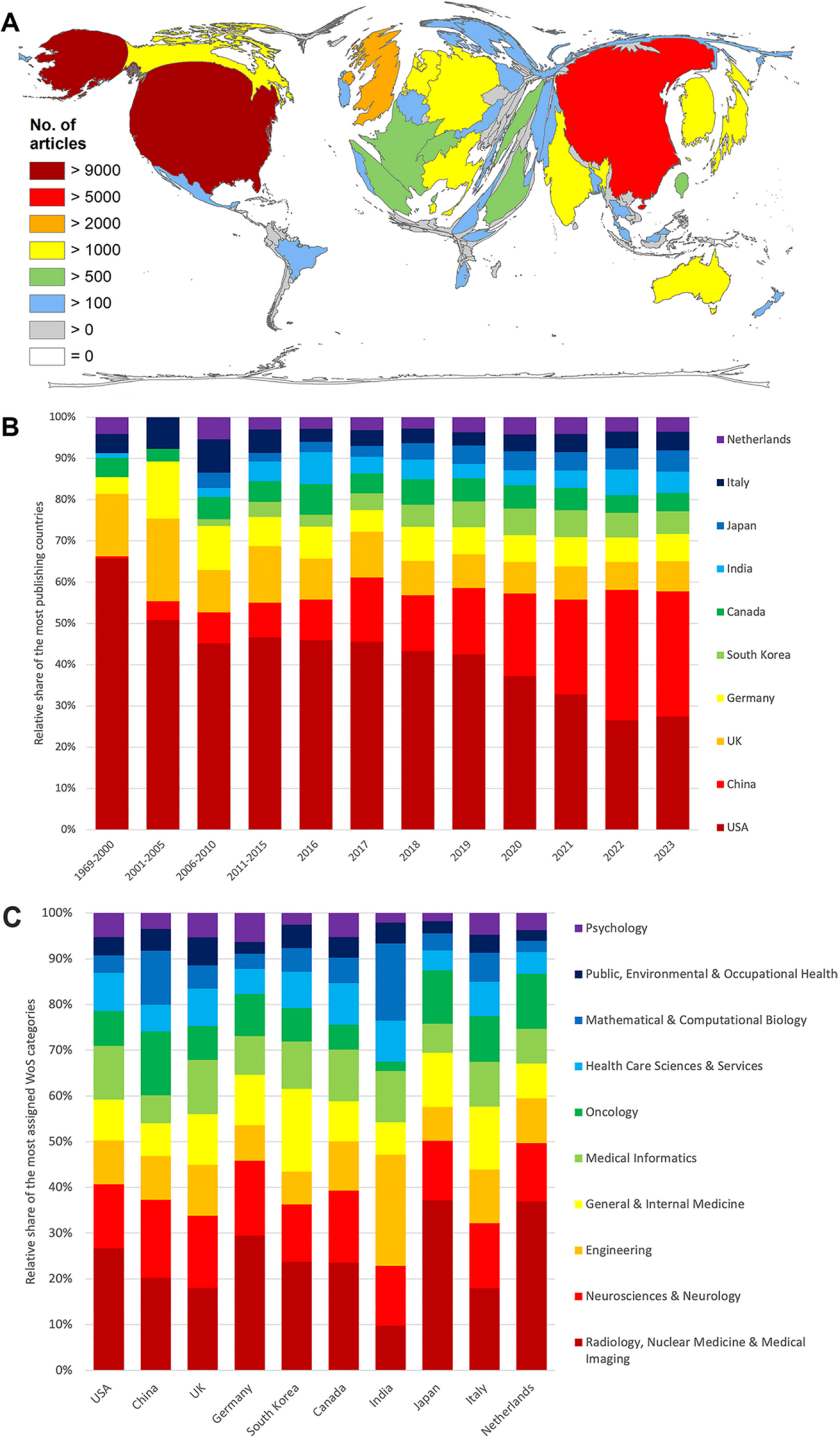
Density equalizing map projections of publication parameters. (**A**) Number of articles on AI_med_. (**B**) Development of countries’ participation on AI_med_ in 5-year intervals and year. (**C**) Relative share of the most assigned Web of Science (WoS) categories of the ten most publishing countries

The main players in AI_med_ publications were from the USA (*n* = 9707) and China (*n* = 7410), with more than four and three times as many articles, respectively, as the following country: the UK (*n* = 2287). Fourth is Germany (*n* = 1971), followed by South Korea (*n* = 1761), to name the five countries with the most publications on AI_med_ (Fig. 3A). The rankings of the most-cited countries are similar (USA: c = 197,032, China: c = 73,298, the UK: c = 48,236, and Germany: c = 37,943); only South Korea (c = 24,785) is pushed down to 7th place by the Netherlands (c = 30,566) and Canada (c = 25,962). In terms of the citation rate (threshold *≥* 30 articles on AI_med_), the Netherlands is in the lead (cr = 27.64), followed by Slovenia (cr = 27.01), Greece (cr = 23.54), Cyprus (cr = 22.89) and Singapore (cr = 21.80) (Suppl. Table 2). Looking at the development over time, it is clear that China has overtaken the USA concerning the number of publications and has been at the top of the ranking since 2022 (Fig. 3B).

Generally, the research foci of the countries differ concerning some topics. The largest proportion of articles from Japan and the Netherlands were from *Radiology*,* Nuclear Medicine & Medical Imaging*. This focus was relatively low addressed in the UK and India. India, in particular, has a greater proportion of publications in the field of *Engineering* and *Mathematical & Computational Biology* (Fig. 3C).

**Fig. 4:**
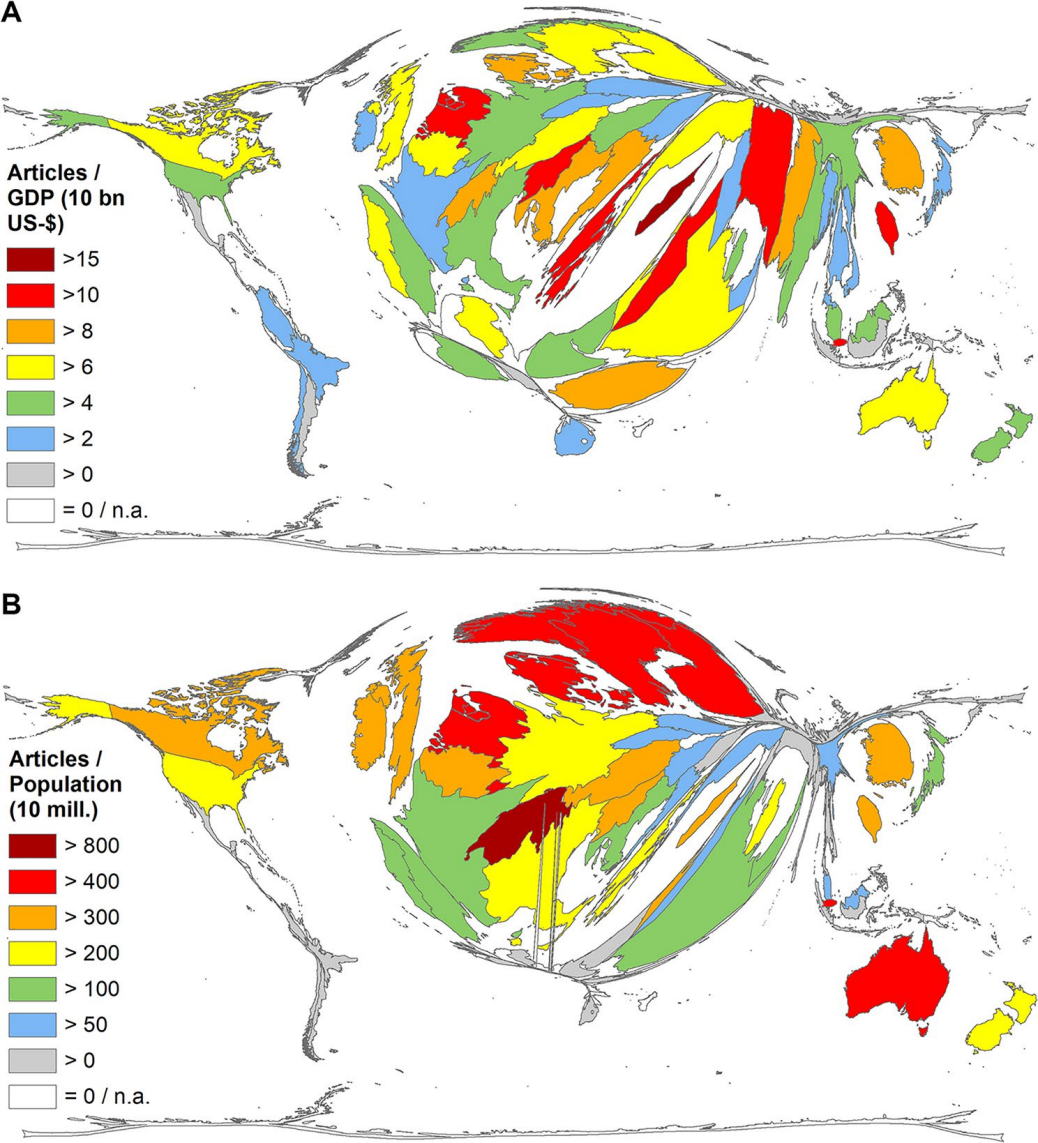
Density equalizing map projections of socio-economic parameters (threshold *≥* 30 Articles on AI_med_). (**A**) Ratio of the number of articles and the gross domestic product (GDP) in 10 billion US-$) (R_GDP_). (**B**) Ratio of the number of articles and the population size in 10 million inhabitants (R_POP_)

Putting the socioeconomic values in relation to the number of articles per country (threshold > 30 articles on AI_med_), other rankings emerge. In terms of R_GDP_ (number of articles/GDP in US$ 10 billion), Cyprus was in the lead (R_GDP_ = 16.59), followed by Jordan (R_GDP_ = 12.38), Iran (R_GDP_ = 11.62), Slovenia (R_GDP_ = 11.54) and Greece (R_GDP_ = 11.51) (Fig. 4A). In terms of R_POP_ (number of articles/population in million inhabitants), Switzerland ranks first (R_POP_ = 899.60), followed by Singapore (R_POP_ = 770.22), the Netherlands (R_POP_ = 644.03), Denmark (R_POP_ = 596.94), and Luxembourg (R_POP_ = 472.44) (Fig. 4B). The leading countries in absolute terms fall sharply in the terms of socioeconomic evaluation.

### Economic and innovativeness influence

**Fig. 5:**
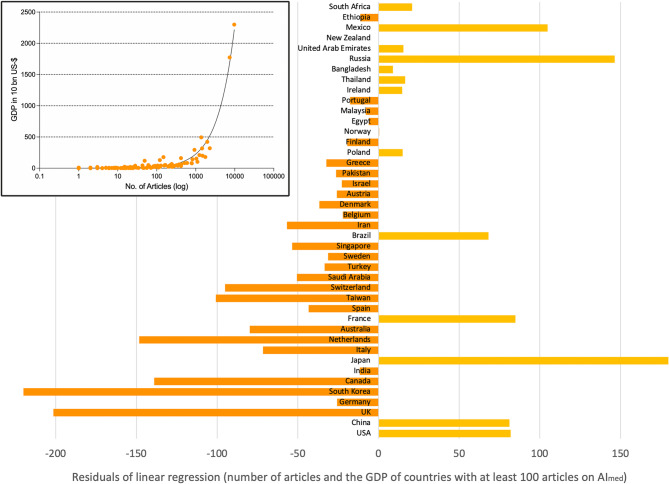
Residuals of the linear regression between number of articles and GDP (in 10 billion US-$) [32] of countries with more than 100 articles on AI_med_. Number of articles per country decreases bottom-up. In the Box: Linear regression (Spearman): r^2^ = 0.97. Correlation significant: *p* < 0.0001. Negative residuals signify values in favor of the number of articles on AI_med_ in relation to GDP per country, and positive residuals to the disadvantage of the number of articles

Correlation analysis (Spearman) revealed significant correlations between the number of articles on AI_med_ and the economic power of the publishing countries (*p* < 0.0001). Looking at the residuals of the linear regression between the two parameters, values emerge that worked in favor (negative residuals) or to the disadvantage (positive residuals) of publishing efforts on AI_med_. Among the countries that publish the most, the USA, China, Japan and France are notable for their high positive residual values. This means a relatively low publication output in relation to their economic power. In contrast, the UK and South Korea, in particular, show favorable performance (Fig. 5).

### Publication trends

**Fig. 6:**
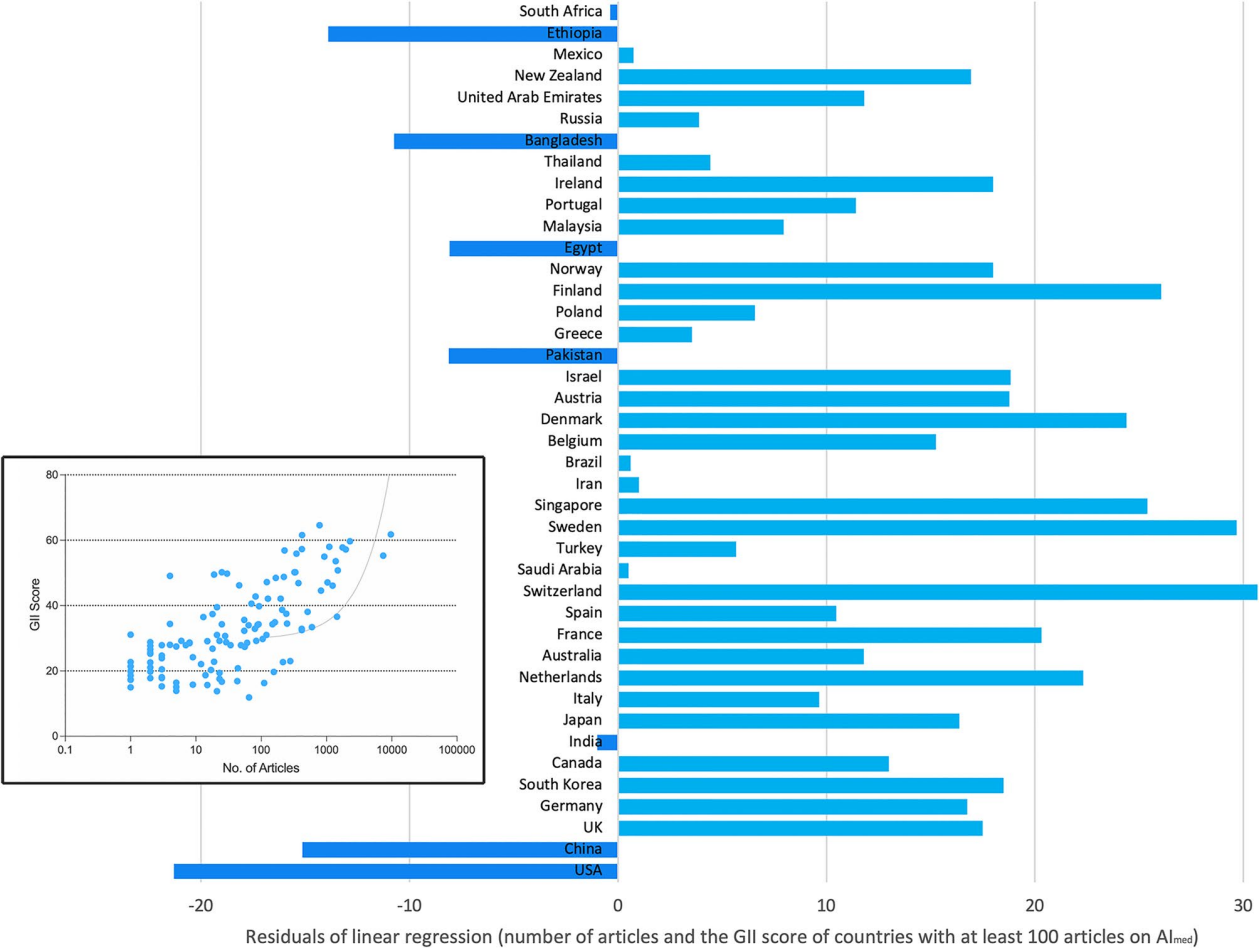
Residuals of the linear regression between the number of articles and the GII score [34] of countries with more than 100 articles on AI_med_. Number of articles per country decreases bottom-up. In the box: Linear regression (Spearman): r^2^ = 0.20. Correlation significant: *p* < 0.0001. Negative residuals signify values in favor of the number of articles on AI_med_ in relation to the GII score per country, and positive residuals to the disadvantage of the number of articles

The rankings of the countries with the most publications on AI_med_ (*n* > 100) sorted by the countries’ degree of innovation (GII) are shown in Supplementary Table 3. The correlation between the GII score and the number of articles on AI_med_ was also significant (*p* < 0.0001). However, the residuals of the linear regression show other tendencies for the most-publishing countries. Here, the USA and China show a favorable relationship concerning GII and the number of articles on AI_med_ together with Pakistan, Egypt, Bangladesh, and Ethiopia. The UK, Germany, and South Korea perform more negatively (Fig. 6). The correlation between the number of articles and the readiness for AI usage (GAIRI score) was also significant (*p* < 0.0001). The deviation from the regression line is similar to that of the GII residuals, with the USA and China also leading in the number of items and AI together with Egypt, Bangladesh, and Ethiopia. Only Pakistan has a less favorable position in this context (Supp. Figure 2).

### Affiliations

**Fig. 7:**
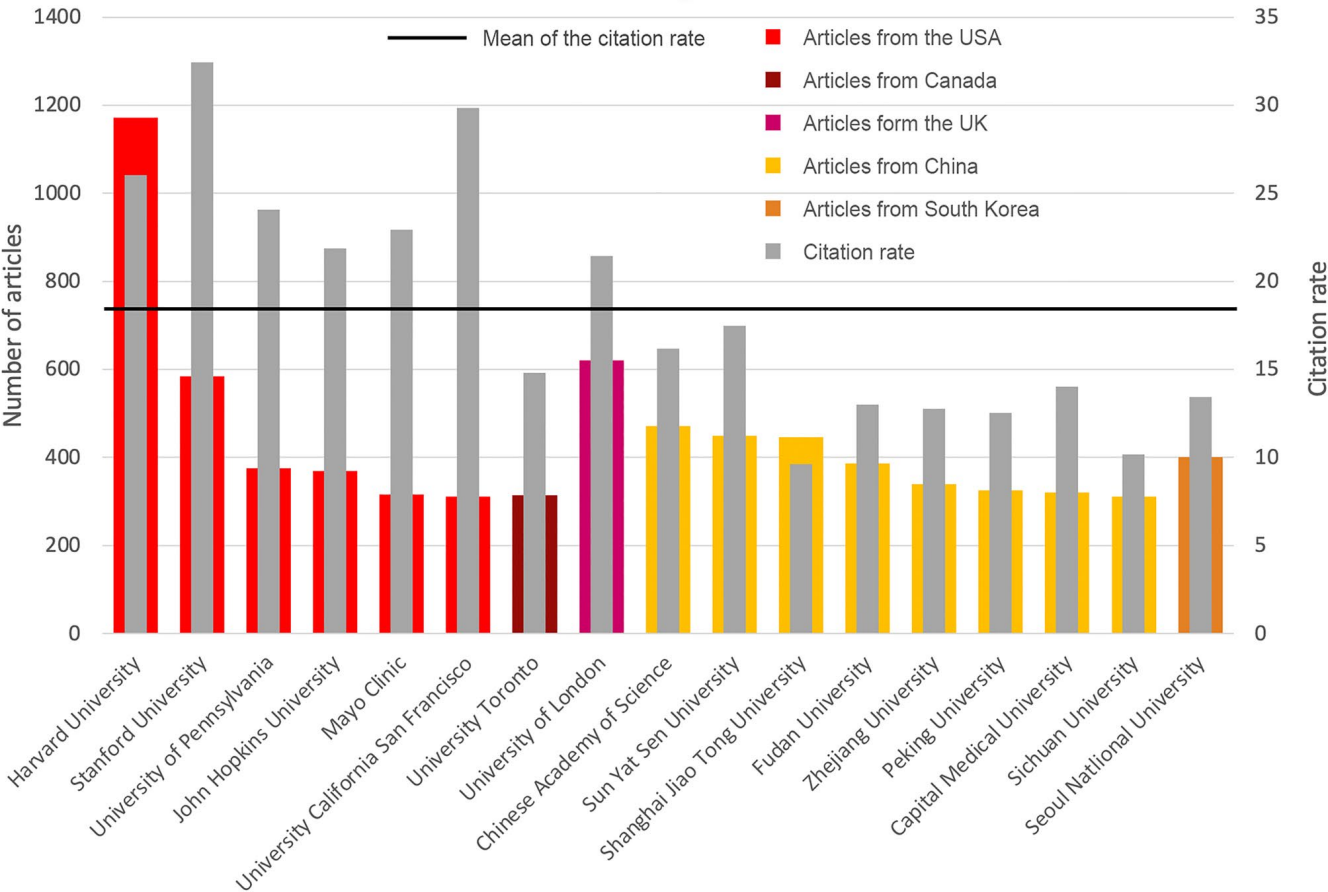
Number of articles and average citation rate of the most publishing institutions with more than 300 articles on AI_med_

The US Harvard University is the scientific institution with the most publications on AI_med_ (*n* = 1171), followed by the University of London (UK) (*n* = 620), Stanford University (USA) (*n* = 585), the Chinese Academy of Science (CAS) (*n* = 471), and Sun Yat-Sen University, which are also from China (*n* = 449). While Asian institutions can keep up with US or UK institutions concerning the number of articles, they lose out at citation counts. Compared to the mean citation rates of the most frequently publishing institutions, Asian institutions received fewer citations, while US and EU institutions received more citations (except for the Canadian University of Toronto, which has achieved a citation rate that is also below the mean) (Fig. 7).

Table 3 lists the private companies that publish most frequently on AI_med_.

**Table 3:** Ten most publishing companies in aimed

Company	Headquarters	Articles
Siemens	Germany	285
GE	USA	164
Philips	Netherlands	126
Google	USA	91
IBM	USA	91
Canon Inc	Japan	70
AstraZeneca	UK, Sweden	65
Novartis	Switzerland	65
Ping An Good Doctor	China	52
AtheroPoint	USA	51

### Collaborations

In total, *n* = 8633 collaborations between at least two partner countries were elaborated on AI_med_ (29.57% of all articles). Of these, *n* = 2710 articles were published in 2022, following a similar increase as all the articles (29.40% of all the articles published in 2022), thus, the percentage of collaboration articles has remained the same over the years. Most collaborations on AI_med_ are binational (67.42%). Only *n* = 72 articles were co-authored by ten or more countries (0.83%).

**Fig. 8:**
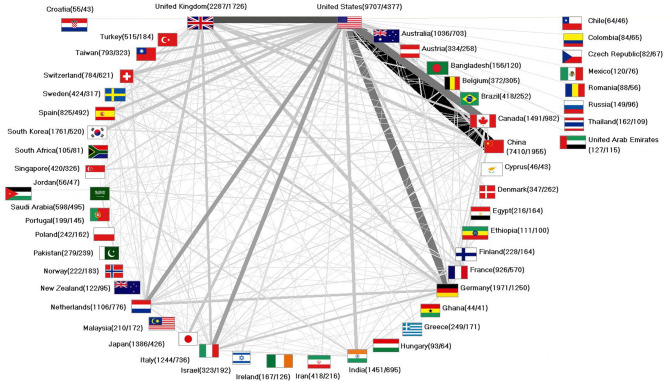
International collaboration network (threshold for display: 25 collaborations from at least two countries). Numbers in brackets (articles on AI_med_ / collaboration articles). The strength of the connecting lines symbolizes the intensity of the collaboration

The USA is at the center of international collaboration. The most international collaborations on AI_med_ took place between the USA and China (*n* = 1054), followed by a distinctly lower number by the USA and the UK (*n* = 670). Collaboration between the USA and Canada resulted in *n* = 567 articles. The cooperation with Germany resulted in *n* = 561, and with Italy in *n* = 331 articles. The share of international cooperation of North American or EU countries is between the shares of Asian countries and the share of African countries, which have the highest shares in comparison (Fig. 8):

1. North America / EU: USA (45.09%), UK (75.47%), Germany (63.42%), Canada (65.59%).
2. South East Asia: China (26.38%), South Korea (35.21%), India (47.90%), and Japan (30.74%).
3. Africa: Egypt (75.93%), Ethiopia (90.01%), South Africa (77.14%).

In terms of the number of articles from a single country, China (*n* = 5455) was leading, followed by the USA (*n* = 5455), South Korea (*n* = 1241), Japan (*n* = 9609), and India (*n* = 756).

## Discussion

Several bibliometric analyses have already been carried out, focusing on AI in medicine. In most cases, however, these relate to a specific medical topic [36–38] but not to the field of AI in medicine and healthcare as a whole. One study was conducted on AI_med_. It only extended to 2018 and provided a first look at keywords, authors, and countries of origin [39]. The novelty of this study is, therefore, that it paints a comprehensive picture of global research on AI_med_, interpreting socio-economic characteristics and, thus, highlighting particular country-specific research incentives and constraints.

### Chronological aspects

The chronological analysis of global publication development shows connections with the history and development of AI_med_. After a phase of initial enthusiasm about the possibilities of AI, disillusionment followed in the 1970s. In reference to the term “nuclear winter” from the Cold War era, the “AI winter” refers to the period in which AI activity in business and academia declined dramatically after the US government decided to scale back its involvement in AI research [40]. The previously overestimated promises of AI have not been fulfilled during this time. The 1973 *Lighthill Report* [41], initiated by the British Research Council, did its part by stating that no area of AI had had the major impact promised. Research output held correspondingly low during this period.

Nevertheless, AI did not find its way into medicine or the health sciences until the 1970s [42]. The first medical approaches published in WoS were developed to facilitate antimicrobial therapy for physicians [43] and pattern recognition in neurobiology [44]. In the 1980s and 1990s, little research was conducted that focused on faster data collection, surgical intervention support, *database administration* (DBA), and electronic health record (EHR) implementation [42].

The low level of annual numbers changed with the beginning of the 2000s, when research related to AI_med_ first increased slowly, followed by a sharp increase in 2017. Advances in the function of AI in medicine led to this development. The most cited articles on AI_med_ were published during this time, showing a strong interest in AI_med_ [45–47]. They are related to diagnosis with AI in medical imaging algorithms, gene expression profiling, and DL with convolutional neural networks. Before this sharp increase, another peak in the annual citation rate occurs. The year 2009 presented no highly cited articles but some well-recognized studies on pattern recognition of ML in MRI [48, 49]. However, the annual citation numbers started already to rise rapidly before the increase in publication numbers. Until now, they reached their highest annual value in 2020, when the most frequently cited articles were published. Since publications in the biomedical sciences usually take approximately eight years to achieve half of their citation (*cited half-life*: CHL) [50], this development underlines the extremely strong interest in the AI_med_ articles with a certainly increasing future trend.

However, the development of AI has been associated with high risks and problems, particularly in the field of medicine. In recent years, AI has also been used to create fake news on medical topics, creating and spreading confusion. This happened, for example, in the manipulation of people, which led to a worldwide movement against vaccinations [51]. The EU AI Act, which was introduced in 2024, was the first instrument to legally regulate AI and thus respond to the growing and irrefutable concerns and fears, including on the professional side [2].

### Aspects of research foci

Similarly, *Radiology/Nuclear Medicine* is the most frequently addressed research focus for title and keyword analysis, demonstrating the importance of AI for the application of medical image patterns. Not surprisingly, the WoS categories *Oncology*, *Cardiology*, and *Neurology* are also very commonly addressed in AI_med_. The prediction of mortality and morbidity is an important topic for genetics studies and is also underscored by frequently occurring terms. Cluster analyses revealed the deep interconnectedness of research patterns around the concepts of AI, ML, and DL.

The research foci of the publishing countries are mostly similarly distributed.

Comparing the two leading countries, AI research in China– in contrast to that in the USA– is more dominated by mathematical and computational aspects. China is also slightly stronger in articles on oncology, while its share of articles on medical imaging is lower. These two countries are by far the most active in the field of AI_med_.

### Geographical aspects

In line with the former studies [36–38], the USA and China published the most on AI_med_, followed by some European countries. While Italy ranks among the top five countries in these previous studies, it ranks only 9th in our analysis. Italy’s share has decreased since 2016 when other European countries were doing more research on AI_med_.

Until the 2000s, the USA was the absolute leader. China has been gradually catching up since then and has overtaken the USA since the 2020s. In terms of the number of citations, China is still far behind, with approximately 37% of the citations for US articles at the time of the evaluation. This is certainly due in small part to the higher proportion of mathematical and computational foci in Chinese articles, as these disciplines are generally cited comparatively less when looking at the Journal Citation Report (JCR) of the journals in question. However, this cannot be the sole reason for the lower citation numbers. China is also not involved in the most cited articles and ranks only 18th with its most cited study as a first-author country with a 2020 study on AI for COVID-19 detection [52]. This lower citation level is also underscored by the comparison between US and Chinese institutions. China has certainly gained a lot with its 2017 *New Generation AI Development Plan* [53]. This plan focuses primarily on promoting AI development but also initiates a high-level time frame for regulations, e.g., the Cybersecurity Law of 2017 [54]. Its introduction has led to a large increase in the number of related scientific studies in China. However, China also needs to pay attention to the quality of its projects. Only future analysis will show whether this plan has been or will be successful, both in terms of global recognition of scientific studies as measured by the number of citations. Regulatory principles, such as the guidelines for the registration and review of AI-based medical devices published by the National Medical Products Agency (NMPA) in 2022 [55], must prove their worth. It is said that policymakers in other countries can learn from these plans as they give a sneak of what might be possible and what might be futile. It must be taken into account that this development has its roots in China’s regulation of internet content, which illustrates the CCP’s (*China Communist Party*) political interest. Therefore, few actors will swim against the ideological tide, and opposing solutions will not be considered [53]. In comparison, there is currently no specific regulatory pathway for AI technologies in the USA. Here, the *Food and Drug Administration* (FDA) evaluates them under the framework conditions for other medical devices [56]. In addition, the EU and Brazil are attempting to regulate the development of AI on a risk-based basis, while most other countries are seeking national guidelines for AI applications in healthcare [55].

### Economic and equity aspects

As we have shown, the level of economic power, innovation, and readiness for AI is significantly correlated with publication performance on AI_med_ worldwide. Previous studies on a variety of scientific topics have also shown that the economic strength of countries is linked to their publication performance [57–59]. Therefore, developing countries or countries with lower economic power are underrepresented in AI_med_ research. However, some of these countries have been able to perform well in terms of publication numbers, albeit at relatively low scores. These countries include Pakistan, Egypt, Bangladesh, and Ethiopia. Despite their low economic power, these countries can be singled out as examples of having good opportunities for AI development in the health sector. Fundamentally, countries in the Global South are in a worse position to benefit from AI technologies because they do not have the opportunity for equal progress and implementation. This may exacerbate global inequalities in the health sector as a whole [35]. In addition to Cyprus, which is in the lead with respect to the R_GDP_ analysis. Also, Jordan and Iran are among the top three ranks. Both countries are categorized as LMI economies [60].

Differences concerning the country of origin can also be shown for the research foci. India, for example, is the only country in the top ten that is not an HI economy. It has the lowest proportion of articles on medical imaging, but a higher proportion of articles on math, computing and engineering topics compared to the other top ten countries. These focus areas also refer to Jordan, Iran, and Pakistan’s foremost themes, engineering and mathematics. In contrast to Jordan and Pakistan, which mostly collaborate with Saudi Arabia, Iran is more scientifically linked to Western countries (the USA and Canada). India collaborates most frequently with the USA, but this is immediately followed by a collaboration with Saudi Arabia, which shows that this is an important scientific partner in AI_med_ research in these parts of the world. With Saudi Arabia’s comparable low citation rate, collaborations with Western HI countries lead to a higher reputation in the scientific community.

The differences between countries in terms of performance and readiness in AI are due to structural characteristics in infrastructure and research [61]. This is driven by different levels of development and funding, particularly in AI_med_, which is additionally dependent on well-curated data [18, 39].

### Affiliation aspects

This difference in the global recognition of research output on AI_med_ is also reflected in the metrics of the publishing institutions, which also show an inferiority of the Global South with significantly lower citation figures compared to Western countries. Despite its participation in highly cited papers [62, 63], the Canadian University of Toronto achieves values well below the average of the ten leading institutions. It is, therefore, an exception in terms of the citation frequency of AI_med_ publications. This may be due to the late increase in the use of its research in AI_med_ (2019), thus, better citation values can be expected in the future.

In contrast to general AI research, private companies are less involved in AI_med_ [64]. The US company Google LLC ranks first in general AI, even when looking at the joint ranking of science and industry. Google lost its pole place in AI_med_, where it is ranked behind Siemens Corp (Germany), GE Healthcare (USA), and Philips Corp (UK). In the area of general AI research, Microsoft is in second place, followed by Facebook. GE Healthcare, which is frequently active in AI_med_, is not included in the general AI list.

A comparison of the countries’ research performance on general AI and AI_med_ shows– similar to industrial research– that Germany is more active in the medical context. Instead, Israel is more involved in general AI, where it ranks third [65].

### Future aspects

Countries around the world are currently striving to equip their scientific, bureaucratic, and industrial systems with AI capabilities, not only to exploit their potential but also to take a leading position in further development. Therefore, the next few years will show who will set the standards. However, the steps have been mapped out, and the risks cannot be denied, thus, most countries have already developed and will continue to develop strategies to tackle the problem [66].

The WHO has already emphasized the need to promote international cooperation in the field of AI governance, especially with LMI countries. Algorithms based on data from HI countries are often not applicable to LMI environments [23] and current regulations are often inadequate. Ethical governance of health data at the global level and regionally focused impact assessment must be prioritized [24]. To avoid a regional concentration of AI technologies in HI countries, which deprives a large part of the world, the cross-border flow of data must be ensured [67].

## Conclusions

However, crucial challenges remain, and the need for standardized, secure, ethical, and transparent solutions is as unwavering as the exorbitant risks of AI_med_ remain. In the interest of global health equity, underserved regions must be supported and their epidemiological and socioeconomic data must be included in the development processes of the global AI network on health issues. Therefore, LMI countries in particular must be supported with financial, technological, and human resources and networking so that medical AI techniques take into account their psychological and physiological characteristics as well as their very own points of view. Joint multi-stakeholder engagement and new approaches to security and transparency throughout the lifecycle of AI_med_ technology can help. Training programs and infrastructure projects must be carried out at the international level with the participation of developing countries to create global qualifications and capacities.

Nevertheless, the following question remains: Is the safe and non-discriminatory use of AI in medicine still feasible at all from this point in the development of such a broad range of possible applications? This challenge should be at the heart of future global scientific engagement, with a focus on clinical, ethical, and technical robustness.

## Electronic supplementary material

Below is the link to the electronic supplementary material.

Supplementary Material 1

## Data Availability

The datasets used and/or analyzed during the current study are available from the corresponding author upon reasonable request.

## Abbreviations

AI: Artificial Intelligence
AI_med_: Artificial Intelligence in the field of medicine
CAIS: Center for Artificial Intelligence Safety
CAS: Chinese Academy of Science
CHL: Cited Half-life
CT: Computer Tomography
DBA: Database Administration
DL: Deep Learning
DEMP: Density equalizing map projections
EHR: Electronic health record
EU: European Union
FDA: Food and Drug Administration (USA)
FLI: Future of Life Institute
GAIRI: Governmental AI Readiness Index
GDP: Gross Domestic Product
GII: Global Innovation Index
HI country: High-income country
IRB: Institutional Review Board
JAMA: Journal of the American Medical Association
JCR: Journal Citation Report
LAWS: Lethal Autonomous Weapon Systems
LMI country: Low- or middle-income country
LMM: Large Multimodal Models
MRI: Magnetic Resonance Imaging
MEP: Members of the European Parliament
ML: Machine Learning
NewQIS: New Quality and Quantity Indices in Science
NMPA: National Medical Products Agency (China)
REC: Research ethics committee
UIS: UNESCO Institute for Statistics
UN: United Nations
WIPO: World Intellectual Property Organization
WoS: Web of Science
