# Supplementary material for: Research on artificial intelligence, machine and deep learning in medicine: global characteristics, readiness, and equity
Source: Global Health. 2025 Jun 8;21:36. doi: 10.1186/s12992-025-01128-1 (PMC12147299; doi:10.1186/s12992-025-01128-1)

**Supplement for**

**Research on the Contribution of Artificial Intelligence, Machine and Deep Learning in Medicine: Global Characteristics, Readiness, and Equity**

Doris Klingelhöfer, Markus Braun, Janis Dröge, David A. Groneberg, Dörthe Brüggmann

**Supplement Tables:**

Supplement Table 1: Included Web of Science Categories, sorted by the number of assigned articles.

- Environmental Sciences
- Public Environmental Occupational Health
- Environmental Studies or Multidisciplinary Sciences
- Engineering Environmental or Water Resources
- Meteorology Atmospheric Sciences or Biology or Oncology
- Toxicology or Green Sustainable Science Technology
- Medicine General Internal or Ecology
- Marine Freshwater Biology or Genetics Heredity
- Oceanography or Soil Science or Endocrinology Metabolism
- Plant Sciences or Food Science Technology
- Medicine Research Experimental
- Psychiatry or Psychology Multidisciplinary
- Pharmacology Pharmacy or Geology or Hematology
- Fisheries or Psychology Applied or Zoology or Clinical Neurology
- Limnology or Veterinary Sciences or Psychology
- Pediatrics or Pathology or Health Care Sciences Services
- Surgery or Neurosciences or Forestry
- Biodiversity Conservation or Agriculture Multidisciplinary
- Psychology Social or Physiology or Parasitology
- Evolutionary Biology
- Cardiac Cardiovascular Systems
- Entomology
- Health Policy Services
- Agriculture Dairy Animal Science
- Nutrition Dietetics or Tropical Medicine
- Obstetrics Gynecology or Psychology Clinical
- Immunology or Behavioral Sciences
- Emergency Medicine
- Urology Nephrology
- Psychology Experimental
- Nursing or Infectious Diseases
- Geriatrics Gerontology
- Otorhinolaryngology
- Peripheral Vascular Disease
- Reproductive Biology
- Gastroenterology Hepatology
- Respiratory System
- Dentistry Oral Surgery Medicine
- Gerontology
- Horticulture
- Psychology Developmental
- Dermatology
- Developmental Biology
- Psychology Educational
- Orthopedics
- Virology
- Ophthalmology
- Ornithology
- Psychology Biological
- Medicine Legal
- Audiology Speech Language Pathology
- Transplantation
- Anesthesiology
- Critical Care Medicine
- Medical Ethics
- Psychology Psychoanalysis
- Rehabilitation
- Allergy
- Anatomy Morphology
- Integrative Complementary Medicine
- Primary Health Care
- Rheumatology
- Substance Abuse
- Neuroimaging

Supplementary Table 2: Citations and Citation rate of countries with at least 100 articles on AI_med_.

| **Country** | **Articles** | **Citations** | **Citation rate** |
| --- | --- | --- | --- |
| Netherlands | 1106 | 30566 | 27.64 |
| Slovenia | 71 | 1918 | 27.01 |
| Greece | 249 | 5862 | 23.54 |
| Cyprus | 46 | 1053 | 22.89 |
| Singapore | 420 | 9157 | 21.80 |
| Argentina | 61 | 1323 | 21.69 |
| UK | 2287 | 48236 | 21.09 |
| Qatar | 80 | 1646 | 20.58 |
| USA | 9707 | 197032 | 20.30 |
| Austria | 334 | 6514 | 19.50 |
| Germany | 1971 | 37943 | 19.25 |
| New Zealand | 122 | 2311 | 18.94 |
| Switzerland | 784 | 14340 | 18.29 |
| Mexico | 120 | 2192 | 18.27 |
| Israel | 323 | 5865 | 18.16 |
| France | 926 | 16542 | 17.86 |
| Sweden | 424 | 7537 | 17.78 |
| Denmark | 347 | 6102 | 17.59 |
| Canada | 1491 | 25962 | 17.41 |
| Hungary | 93 | 1584 | 17.03 |
| Australia | 1036 | 17417 | 16.81 |
| Romania | 88 | 1420 | 16.14 |
| Norway | 222 | 3576 | 16.11 |
| Vietnam | 91 | 1465 | 16.10 |
| Belgium | 372 | 5857 | 15.74 |
| Ireland | 167 | 2625 | 15.72 |
| Italy | 1244 | 19167 | 15.41 |
| Croatia | 55 | 819 | 14.89 |
| Portugal | 199 | 2936 | 14.75 |
| Finland | 228 | 3213 | 14.09 |

Supplementary Table 3: GII (Global Innovation Index), GAIRI (Governmental AI Readiness Index) scores and number of articles on AImed of countries with at least 100 articles on AI_med_.

| **Country** | **GII**  **Score** | **Articles** | **Country** | **GAIRI Score** | **Articles** |
| --- | --- | --- | --- | --- | --- |
| Switzerland | 64.6 | 784 | Singapore | 9.186 | 420 |
| USA | 61.8 | 9707 | UK | 9.069 | 2287 |
| Sweden | 61.6 | 424 | Germany | 8.81 | 1971 |
| UK | 59.7 | 2287 | USA | 8.804 | 9707 |
| Netherlands | 58 | 1106 | Finland | 8.772 | 228 |
| South Korea | 57.8 | 1761 | Canada | 8.674 | 1491 |
| Singapore | 57.3 | 420 | Sweden | 8.674 | 424 |
| Germany | 57.2 | 1971 | France | 8.608 | 926 |
| Finland | 56.9 | 228 | Denmark | 8.601 | 347 |
| Denmark | 55.9 | 347 | Japan | 8.582 | 1386 |
| China | 55.3 | 7410 | Australia | 8.126 | 1036 |
| France | 55 | 926 | Norway | 8.079 | 222 |
| Japan | 53.6 | 1386 | New Zealand | 7.876 | 122 |
| Canada | 50.8 | 1491 | Netherlands | 7.659 | 1106 |
| Austria | 50.2 | 334 | Italy | 7.533 | 1244 |
| Israel | 50.2 | 323 | Austria | 7.527 | 334 |
| Norway | 48.8 | 222 | India | 7.515 | 1451 |
| Ireland | 48.5 | 167 | Switzerland | 7.461 | 784 |
| New Zealand | 47.2 | 122 | UAE | 7.445 | 127 |
| Australia | 47.1 | 1036 | China | 7.37 | 7410 |
| Belgium | 46.9 | 372 | Israel | 7.348 | 323 |
| Italy | 46.1 | 1244 | Malaysia | 7.108 | 210 |
| Spain | 44.6 | 825 | Belgium | 6.859 | 372 |
| Portugal | 42.1 | 199 | South Korea | 6.839 | 1761 |
| UAE | 42.1 | 127 | Poland | 6.835 | 242 |
| Malaysia | 38.7 | 210 | Russia | 6.748 | 149 |
| Turkey | 38.1 | 515 | Portugal | 6.693 | 199 |
| Poland | 37.5 | 242 | Mexico | 6.664 | 120 |
| India | 36.6 | 1451 | Ireland | 6.542 | 167 |
| Thailand | 34.9 | 162 | Spain | 6.332 | 825 |
| Greece | 34.5 | 249 | Brazil | 6.157 | 418 |
| Russia | 34.3 | 149 | Turkey | 5.879 | 515 |
| Saudi Arabia | 33.4 | 598 | Greece | 5.76 | 249 |
| Iran | 32.9 | 418 | Thailand | 5.458 | 162 |
| Brazil | 32.5 | 418 | South Africa | 5.152 | 105 |
| Mexico | 31 | 120 | Iran | 5.049 | 418 |
| South Africa | 29.8 | 105 | Saudi Arabia | 4.779 | 598 |
| Pakistan | 23 | 279 | Pakistan | 4.57 | 279 |
| Egypt | 22.7 | 216 | Bangladesh | 3.808 | 156 |
| Bangladesh | 19.7 | 156 | Egypt | 3.492 | 216 |
| Ethiopia | 16.3 | 111 | Ethiopia | 2.777 | 111 |

**Supplement Figures:**

Supplement Figure 1: Generation of study database. *The common abbreviation AI was additionally searched in the title of the article in combination with the TOPIC (title, abstract, keywords) search "Artificial Intelligence". ** List of Web of Science Core Collection (WoS) categories (Suppl. Table 1).


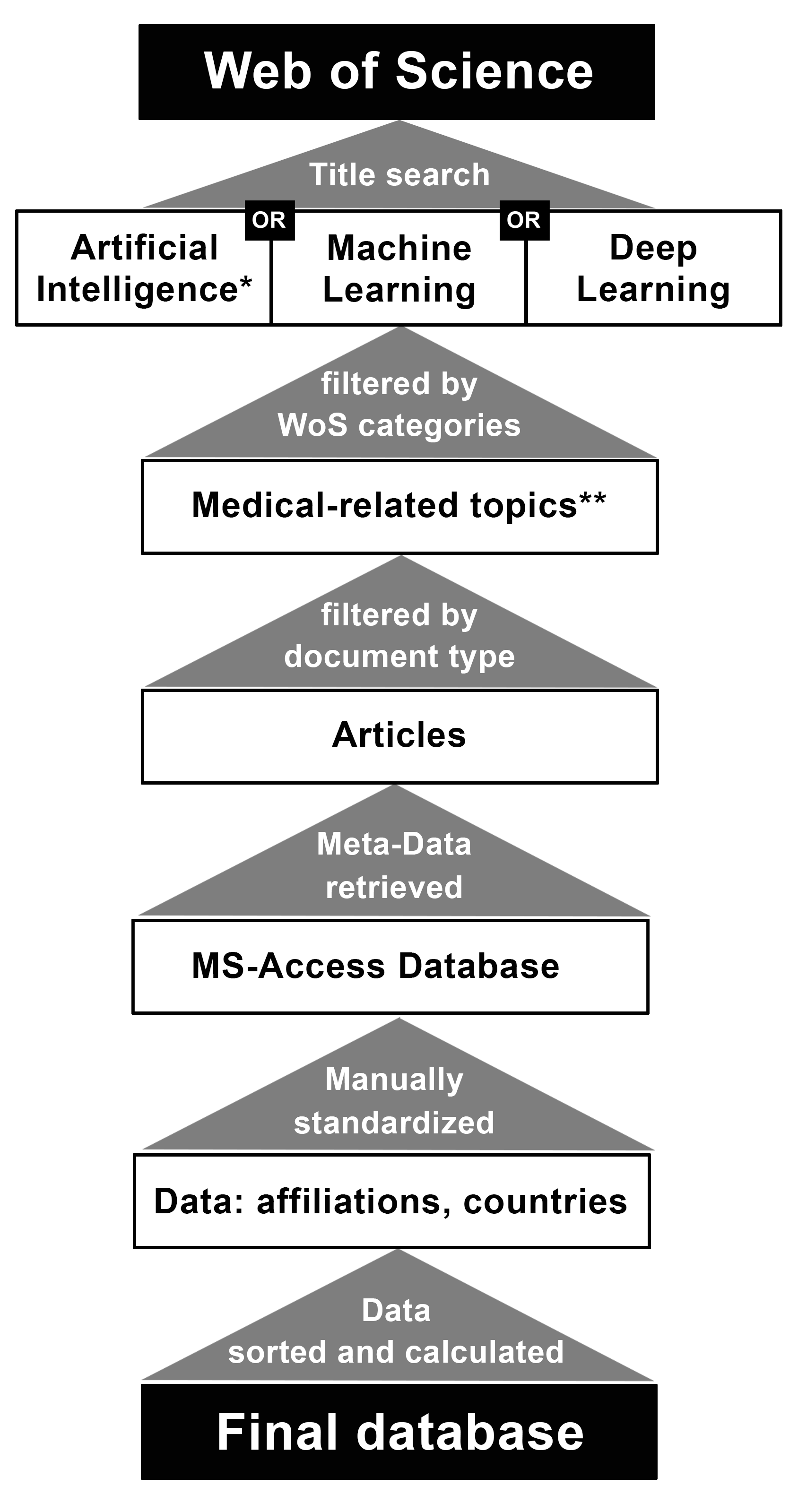


Supplementary Figure 2: Residuals of linear regression between the number of articles on AImed and the GAIRI (Governmental AI Readiness Index) score (threshold: 100 articles on AI_med_ per country).


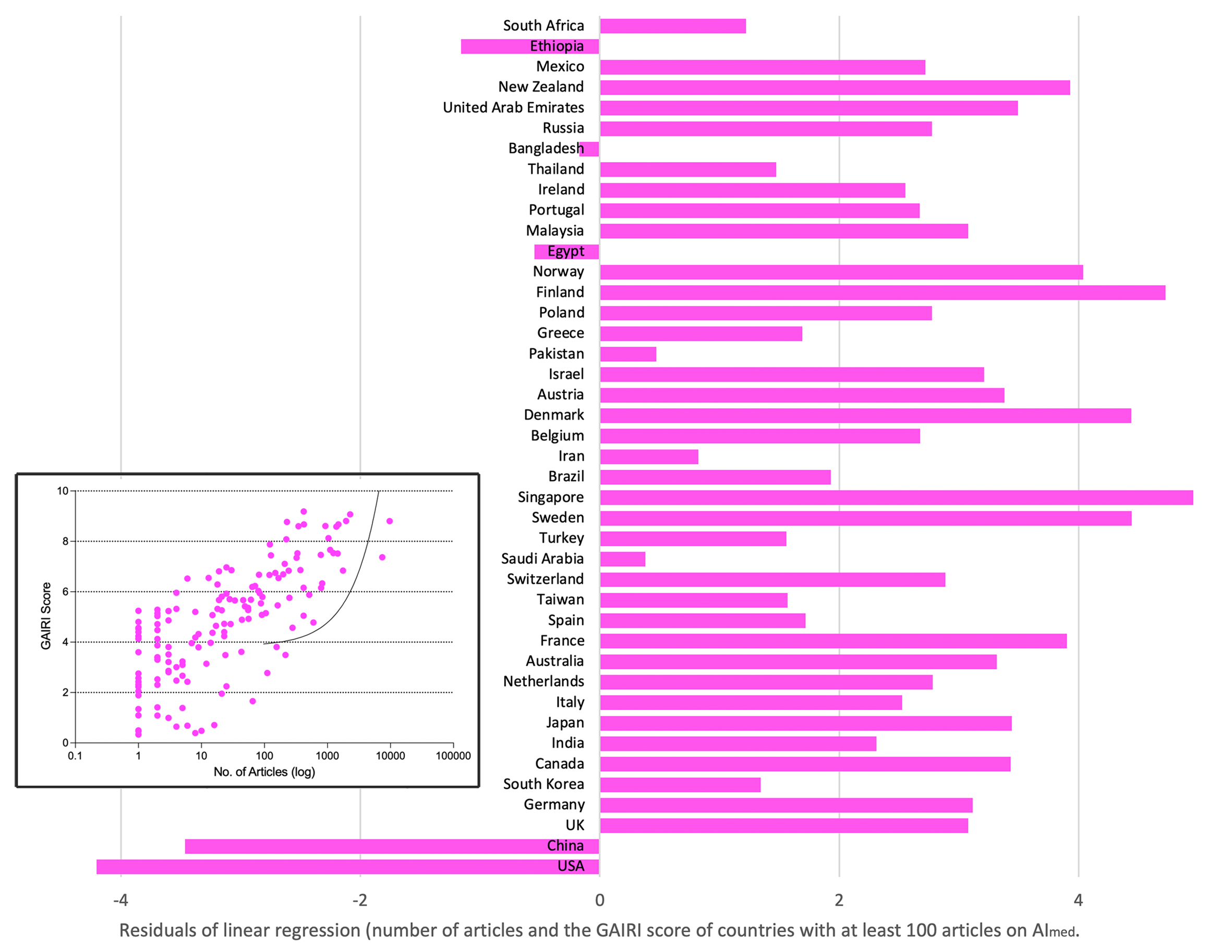

Supplement: Supplementary file 1 — Supplementary Material 1 [file 12992_2025_1128_MOESM1_ESM.docx]
